# Transcriptional study of appetite regulating genes in the brain of zebrafish (*Danio rerio*) with impaired leptin signalling

**DOI:** 10.1038/s41598-019-56779-z

**Published:** 2019-12-27

**Authors:** Ehsan Pashay Ahi, Mathilde Brunel, Emmanouil Tsakoumis, Monika Schmitz

**Affiliations:** 1 Department of Organismal Biology, Comparative Physiology, Evolutionary Biology Centre, Uppsala University, Norbyvägen 18A, SE-752 36 Uppsala, Sweden; 2 Department of Molecular Sciences, Swedish University of Agricultural Sciences, BioCentrum, Allmas Allé 5, SE-750 07 Uppsala, Sweden

**Keywords:** Metabolism, Fat metabolism, Feeding behaviour, Metabolic diseases, Endocrine system and metabolic diseases, Dyslipidaemias, Multihormonal system disorders, Obesity, Transcription, Molecular biology, Physiology, Endocrinology, Neuroscience, Feeding behaviour, Molecular neuroscience, Motivation, Neuronal physiology, Reward

## Abstract

The hormone leptin is a key regulator of body weight, food intake and metabolism. In mammals, leptin acts as an anorexigen and inhibits food intake centrally by affecting the appetite centres in the hypothalamus. In teleost fish, the regulatory connections between leptin and other appetite-regulating genes are largely unknown. In the present study, we used a zebrafish mutant with a loss of function leptin receptor to investigate brain expression patterns of 12 orexigenic and 24 anorexigenic genes under different feeding conditions (normal feeding, 7-day fasting, 2 and 6-hours refeeding). Expression patterns were compared to wild-type zebrafish, in order to identify leptin-dependent differentially expressed genes under different feeding conditions. We provide evidence that the transcription of certain orexigenic and anorexigenic genes is influenced by leptin signalling in the zebrafish brain. We found that the expression of orexigenic genes was not affected by impaired leptin signalling under normal feeding conditions; however, several orexigenic genes showed increased transcription during fasting and refeeding, including *agrp*, *apln*, *galr1a* and *cnr1*. This suggests an inhibitory effect of leptin signal on the transcription of these orexigenic genes during short-term fasting and refeeding in functional zebrafish. Most pronounced effects were observed in the group of anorexigenic genes, where the impairment of leptin signalling resulted in reduced gene expression in several genes, including *cart* family, *crhb*, *gnrh2*, *mc4r*, *pomc* and *spx*, in the control group. This suggests a stimulatory effect of leptin signal on the transcription of these anorexigenic genes under normal feeding condition. In addition, we found multiple gain and loss in expression correlations between the appetite-regulating genes, in zebrafish with impaired leptin signal, suggesting the presence of gene regulatory networks downstream of leptin signal in zebrafish brain. The results provide the first evidence for the effects of leptin signal on the transcription of various appetite-regulating genes in zebrafish brain, under different feeding conditions. Altogether, these transcriptional changes suggest an anorexigenic role for leptin signal, which is likely to be mediated through distinct set of appetite-regulating genes under different feeding conditions.

## Introduction

Feeding behaviour involves complex processes of foraging controlled by appetitive behaviours (hunger-driven activities) and food ingestion^1^. The central regulation of feeding behaviour in the brain is under the influence of endocrine signals, originating from the brain itself, as well as peripheral organs, after exposure to different metabolic and nutritional conditions. The regulation of food intake, in both mammals and fish, takes place in the hypothalamus in the central nervous system. In mammals, appetite regulation through hormones is mediated by their receptors, located on a group of key neurons called arcuate nucleus neurons^2^. In fish, similar neurons exist in the periventricular and lateral parts of the hypothalamus^3^. Nevertheless, it appears that neurons generating neuroendocrine signals display a wider anatomical distribution in the fish brain than in mammals, as it has been observed for appetite-regulating genes expressed in different parts of the fish brain^4^. The neurons mediating the appetite-regulating effects can be divided into two main types: orexigenic, stimulating food intake and/or related locomotor activity; and anorexigenic, reducing food intake and/or related locomotor activity^5^. At the core of these processes are the key molecular players, the so-called appetite-regulation genes, which encode a range of neuropeptides and their cognate receptors. The appetite-regulation genes can also be classified in two categories of orexigenic and anorexigenic genes based on their functions^6,7^. Due to the large diversity of teleost fish in diet, feeding habits, physiology and adaptation to various environmental factors influencing their feeding behaviour, the number of studies addressing molecular mechanisms underlying appetite regulation in fish has been growing in recent years^8^. However, transcriptional activity and regulatory connections between the appetite-regulating genes, under different feeding conditions, are still not fully explored in fish.

One of the signals, transduced by the leptin hormone and its receptor, is an appetite-regulating molecular mechanism, among its other regulatory roles, which include controlling body weight, reproduction, development and stress responses in vertebrates^9,10^. Zebrafish has two leptin hormone genes, *lepa* and *lepb*, which are differently expressed in several organs^11^. Both leptin isoforms mediate their signal through a single leptin receptor gene in zebrafish (*lepr*), which is expressed in several brain regions, including the hypothalamic lateral tuberal nucleus^12^. Most studies of leptin functions in fish are conducted on Cypriniformes (mainly goldfish and zebrafish) and Salmoniformes^8^. Several studies in goldfish showed that excessive level of leptin can reduce both feeding behavior and locomotor activity (anorexigenic effects)^13–16^. The regulatory effects of leptin signal on appetite can be exerted through stimulation of anorexigenic genes, while inhibiting orexigenic genes in the brain^8,13,17,18^. However, the leptin expression in the brain and other organs, in response to different feeding conditions, seems to be highly variable across fish species^8,19^. For instance, post-prandial increase of leptin expression has been observed in goldfish and zebrafish in different organs^16,20^, whereas such an increase was absent in rainbow trout^21^. On the other hand, fasting did not affect the hepatic expression of leptin in goldfish, common carp, pacu and Nile tilapia, while on the contrary, its hepatic expression was affected during fasting in most Perciformes and Salmoniformes examined^8,22^. Furthermore, in zebrafish, inhibiting *lepa* leads to a decrease in metabolic rate^23^, whereas leptin treatment can increase energy expenditure^24^. After feeding the expression of leptin increases in most fishes including goldfish and zebrafish^16,20^. Despite the fact that the number of molecular studies on leptin-dependent regulation of feeding in fish is consistently growing, little is known about regulatory connections between leptin signal and other appetite-regulating genes under different feeding conditions.

To further explore the role of leptin in appetite regulation, we compared in this study the expression levels of 36 genes, which have already been shown in other studies of Cypriniformes to have appetite-regulating functions, *i*.*e*. 12 orexigenic and 24 anorexigenic genes (Table 1), in brain samples of wild-type zebrafish and a zebrafish mutant with a loss of function leptin receptor (*lepr* mutant). We first identified a stably expressed gene in zebrafish brain under different feeding conditions and used it as normalization factor for the analysis of gene expression by qPCR. Next, we aimed to profile the expression dynamics of the appetite-regulating genes during different feeding conditions: normal feeding (control group), fasting and two refeeding stages (2 and 6 hours after refeeding) in wild-type zebrafish and compared the results to the expression patterns observed in the *lepr* mutant. This allowed us to identify genes for which transcriptional changes, in different feeding conditions, may be under the influence of functional leptin signalling. Finally, we predicted loss and gain of potential regulatory connections between the appetite regulating genes in defective leptin signalling,through pairwise expression correlation analysis. Our findings provide comprehensive information about the expression dynamics of known appetite-regulating genes in relation to different feeding conditions, as well as non-functional leptin signalling in the brain of adult zebrafish.

**Table 1:** Descriptions of candidate target genes and their predicted function in appetite regulation.

Genes	Description	Organism	Effects	References
*agrp* (*agrp1*)	Agouti related neuropeptide	Zebrafish	Orexigenic	^35,36^
*apln*, *aplnr*	Apelin, agtrl1 ligand and its receptor	Goldfish	Orexigenic	^30,32^
*cart1-4*	Cocaine and amphetamine regulated transcripts	Zebrafish	Anorexigenic	^43,44^
*cnr1*	Cannabinoid receptor 1	Zebrafish	Orexigenic	^43,108^
*crh*	Corticotropin-releasing hormone	Goldfish	Anorexigenic	^55,56^
*dbi*	Diazepam binding inhibitor, octadecaneuropeptide	Goldfish	Anorexigenic	^109,110^
*galn*, *galr1*,*2*	Galanin/GMAP prepropeptide and its receptors	Goldfish Zebrafish	Orexigenic	^40–42^
*ghrl* *ghsr*	Ghrelin and its receptor (growth hormone secretagogue receptor)	Goldfish Zebrafish	Orexigenic	^111–113^
*gnrh2*,*3* *gnrhr1-4*	Gonadotropin releasing hormone 2 and 3, and their receptors	Goldfish Zebrafish	Anorexigenic	^61,62^
*hcrt*	Orexin, hypocretin neuropeptide precursor	Goldfish Zebrafish	Orexigenic	^90–92^
*kiss1*, *kiss1r*	Prepro-Kisspeptin 1 and its receptor	Sea bass	Anorexigenic	^69^
*mc4r*	Melanocortin 4 receptor	Goldfish	Anorexigenic	^76^
*mch*, *mchr1*,*2*	Pro-melanin-concentrating hormone and its receptors	Goldfish	Anorexigenic	^65,66^
*nmu*	Neuromedin U preproprotein	Goldfish	Anorexigenic	^70,71^
*npy*	Prepro-neuropeptide Y	Goldfish Zebrafish	Orexigenic	^93,94^
*nucb2*	Nucleobindin 2/Nesfatin 1	Goldfish Zebrafish	Anorexigenic	^72–74^
*pacap*	Pituitary adenylate cyclase activating polypeptide	Goldfish	Anorexigenic	^75^
*pomc*	Pro-opiomelanocortin preproprotein	Goldfish Zebrafish	Anorexigenic	^95–97^
*pyy*	Prepro-peptide YY	Goldfish	Anorexigenic	^98^
*scg2*	Secretogranin 2/Secretoneurin	Goldfish	Orexigenic	^99,100^
*spx*	Spexin Hormone	Goldfish Zebrafish	Anorexigenic	^50,51^
*trh*	Thyrotropin-releasing hormone	Goldfish	Orexigenic	^49^

## Results

### Validation of suitable reference gene

Identifying stably expressed reference gene(s) in every specific experimental conditions is a prerequisite for the analysis of relative gene expression levels by qPCR^25–27^. We quantified the expression levels of 8 candidate reference genes in the brains of wild-type and *lepr* mutant, exposed to different feeding conditions. The expression levels of the candidate reference genes varied considerably in the brain of zebrafish with highest expression (lowest Cq) for *actb2*, around 19 average Cq value, to *tmem50a* with lowest expression (highest Cq), around 28 average Cq value (Supplementary Fig. 1A). We found that all the three algorithms, BestKeeper, geNorm and Normfinder, suggested *g6pd* and *rplp0*, as the most and the least stably expressed genes, respectively (Table 2). However, the second and third ranks of the most stable reference genes, were variable for each of the algorithms (the ranks were interchanging between *ef1a*, *rpl13 actb2* and *tmem50a*) (Table 2). Thus, we considered the expression level of only *g6pd* as a normalization factor to quantify the expression levels of our candidate appetite-regulating genes in each sample.

**Table 2:** Expression stability ranking of reference genes in zebrafish brain across wild type and *lepr* mutant adults during the fasting-refeeding experiment.

BestKeeper				geNorm		NormFinder	
Ranking	r values	Ranking	SD values	Ranking	M values	Ranking	S values
*g6pd*	0.676	*g6pd*	0.237	*g6pd*	0.466	*g6pd*	0.124
*ef1a*	0.655	*rpl13*	0.246	*actb2*	0.489	*ef1a*	0.158
*actb2*	0.626	*tmem50a*	0.255	*tmem50a*	0.493	*rpl13*	0.158
*b2m*	0.615	*actb2*	0.268	*rpl13*	0.507	*actb2*	0.161
*rpl13*	0.604	*ube2a*	0.270	*ef1a*	0.524	*tmem50a*	0.169
*tmem50a*	0.531	*ef1a*	0.283	*ube2a*	0.537	*ube2a*	0.173
*ube2a*	0.404	*b2m*	0.418	*b2m*	0.625	*b2m*	0.237
*rplp0*	0.375	*rplp0*	0.577	*rplp0*	0.842	*rplp0*	0.316

Abbreviations: SD = standard deviation, r = Pearson product-moment correlation coefficient, S = stability value, M = mean value of stability.

### Differential expression of orexigenic genes in wild-type versus lepr mutant

**Figure 1:**
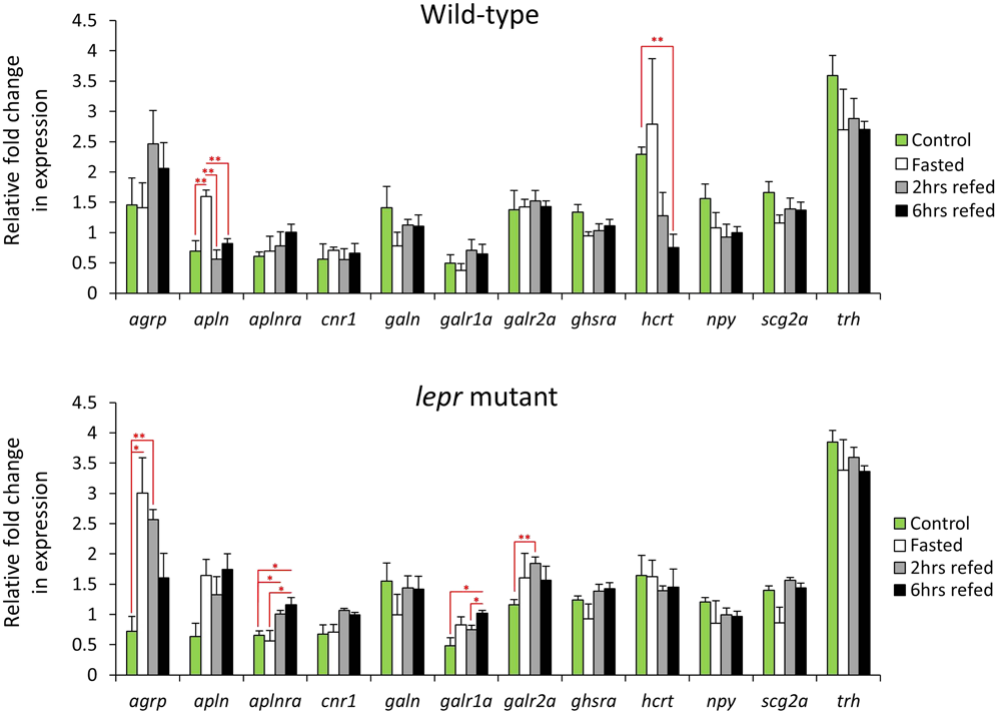
Expression dynamics of orexigenic genes in the brain of wild-type and *lepr* mutant zebrafish during the fasting-refeeding experiment. Means and standard errors of fold changes in expression of five biological replicates are shown for each experimental group. Significant differences between the treatment groups for each genotype are indicated by red asterisks (*P < 0.05; **P < 0.01;).

We investigated the expression levels of 12 orexigenic genes, within wild-type or *lepr* mutant brain during the fasting-refeeding experiment, and found expression changes in a few of the genes for each genotype (Fig. 1). We observed expression differences in wild-type for only 2 genes; *apln* and *hcrt*; whereas in *lepr* mutant, 4 genes showed differences: *agrp* (previously called *agrp1*), *aplnra*, *galr1a* and *galr2a*. Among these DE genes, none of them displayed changes in both genotypes, and even their expression dynamics were not following similar patterns between the genotypes (Fig. 1). These reveal clear differences between wild-type and the genotype with defective leptin signalling in transcriptional regulation of orexigenic genes in brain during fasting and refeeding. We found that one of the DE genes in wild-type zebrafish, *hcrt*, had reduced expression levels in a refeeding group compared to the control, but such a decrease in expression was not observed for any of the DE genes in *lepr* mutant. On the contrary, all of the DE genes in *lepr* mutant displayed increased expression in at least one of the refeeding group compared to the control group, which means stronger appetite stimulation during refeeding when the leptin signalling is impeded (Fig. 1). We also found that *agrp* exhibit an expression induction in *lepr* mutant, and the expression of *apln* is increased in wild-type after fasting. The induced *apln* and *agrp* expressions seem to be the most prominent brain responses among the tested orexigenic genes during fasting in wild-type and *lepr* mutant, respectively.

**Figure 2:**
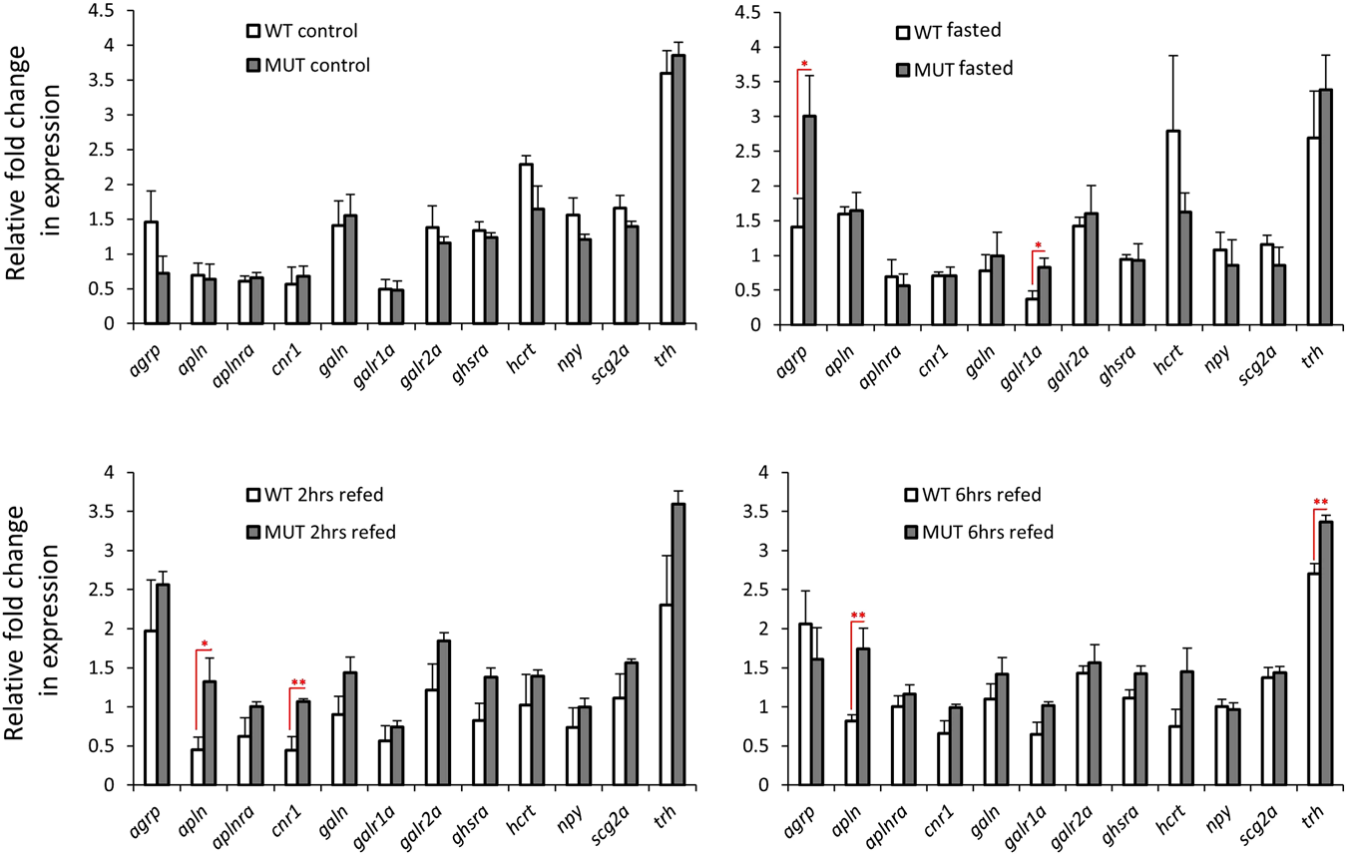
Expression differences of orexigenic genes in the brain of wild-type versus *lepr* mutant zebrafish within each experimental group. Means and standard errors of fold changes in expression of five biological replicates are shown for each experimental group. Significant differences between the wild-type and *lepr* mutant for each treatment are indicated by red asterisks (*P < 0.05; **P < 0.01).

Next, we compared the expression of the orexigenic genes, between wild-type and *lepr* mutant, and did not find any difference in control groups. Thus, under normal feeding conditions, the defective leptin signal does not affect the brain expression of orexigenic genes (Fig. 2). Interestingly, we found *lepr* mutants to have higher expression of several orexigenic genes compared to the control group within the three treatment groups (fasting: *agrp* and *galr1a*; 2 hrs refeeding: *apln* and *cnr1*; 6hrs refeeding: *apln* and *trh*). This shows more appetite stimulation by orexigenic genes, during changes in feeding condition, in defective leptin signalling. Furthermore, we only found one of the genes, *apln*, with increased brain expression in both refeeding groups of *lepr* mutant. Altogether, these findings demonstrate noticeable differences in transcriptional regulation of orexigenic genes in the brain between wild-type and *lepr* mutant strains, under different feeding conditions.

### Differential expression of anorexigenic genes in wild-type versus *lepr* mutant

**Figure 3:**
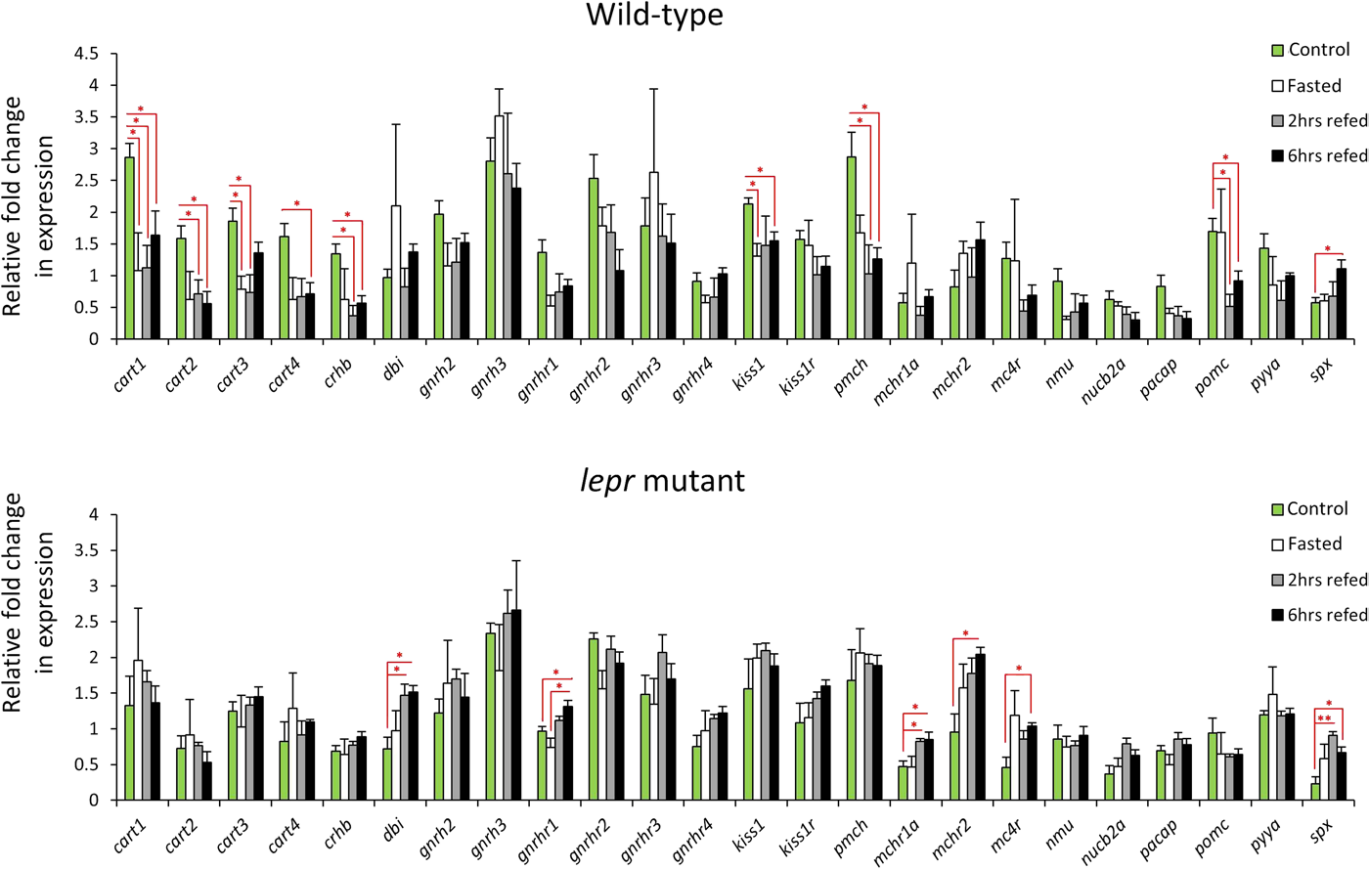
Expression dynamics of anorexigenic genes in the brain of wild-type and *lepr* mutant zebrafish during the fasting-refeeding experiment. Means and standard errors of fold changes in expression of five biological replicates are shown for each experimental group. Significant differences between the treatment groups for each genotype are indicated by red asterisks (*P < 0.05; **P < 0.01).

We investigated the expression levels of 24 anorexigenic genes between wild-type and *lepr* mutant during the fasting-refeeding experiment, and found 15 genes showing expression changes in at least one of the genotypes (Fig. 3). We identified expression differences of 9 genes in the wild-type samples, whereas in *lepr* mutant samples 6 genes displayed differences (Fig. 3). In wild-type zebrafish, all the DE genes, except for *spx*, showed reduced expression in at least one treatment group compared to the control, but all of the DE genes in *lepr* mutant had almost opposite expression patterns with increased expression in at least one treatment group (mainly a refeeding group). Interestingly, we found all *cart* genes displaying similar changes within the wild-type group while no difference was identified in the mutant. *Spx* was the only gene showing similar differential expression pattern in both genotypes; however, it seems that in zebrafish with impaired leptin signal, its induction appeared earlier during refeeding. These findings demonstrate weakened transcriptional regulation for certain anorexigenic genes in functional or defective leptin signalling during the fasting-refeeding experiment (Fig. 3).

**Figure 4:**
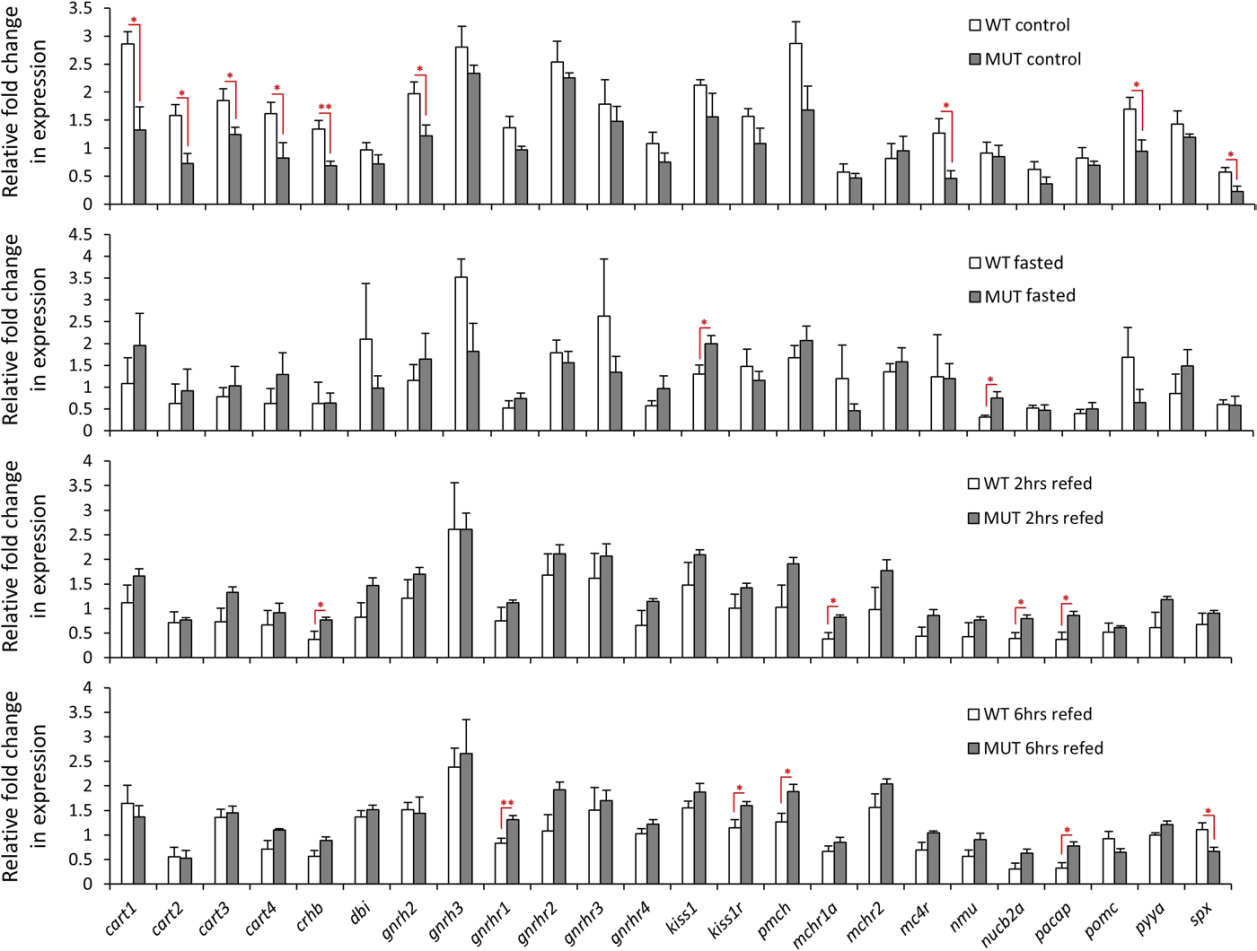
Expression differences of anorexigenic genes in the brain of wild-type versus *lepr* mutant zebrafish within each experimental group. Means and standard errors of fold changes in expression of five biological replicates are shown for each experimental group. Significant differences between the wild-type and *lepr* mutant for each treatment are indicated by red asterisks (*P < 0.05; **P < 0.01).

We directly compared the expression of the anorexigenic genes between the genotypes, and observed expression changes opposite to the orexigenic genes. For instance, while no difference was observed in the control group for orexigenic genes, for the anorexigenic genes, most differences were found in the control group (Fig. 4). All of the DE anorexigenic genes in the control group followed the same pattern of higher expression in wild-type than *lepr* mutant. This indicates that, in contrast to orexigenic genes, defective leptin signal leads to reduction in expression of certain anorexigenic genes, which in turn possibly increases the appetite during normal feeding condition. In the mutant group, we found slight but significant increase in expression of 2 genes during fasting; *kiss1* and *nmu*, 4 genes during 2 hrs refeeding; *crhb*, *mchr1a*, *nucb2a* and *pacap*, and 4 genes during 6 hrs refeeding; *gnrhr1*, *kiss1r*, *mch* and *pacap* (only *spx* had reduced expression in 6 hrs refeeding in the mutant). These findings, during fasting and refeeding conditions, are similar to the orexigenic genes, where only increased expression of the genes was found in the mutant compared to wild-type zebrafish. Therefore, the results demonstrate that functional leptin signalling is required for transcriptional suppression of certain anorexigenic and orexigenic genes, in changes of feeding condition.

### Pairwise expression correlation analyses of the appetite regulating genes

**Figure 5:**
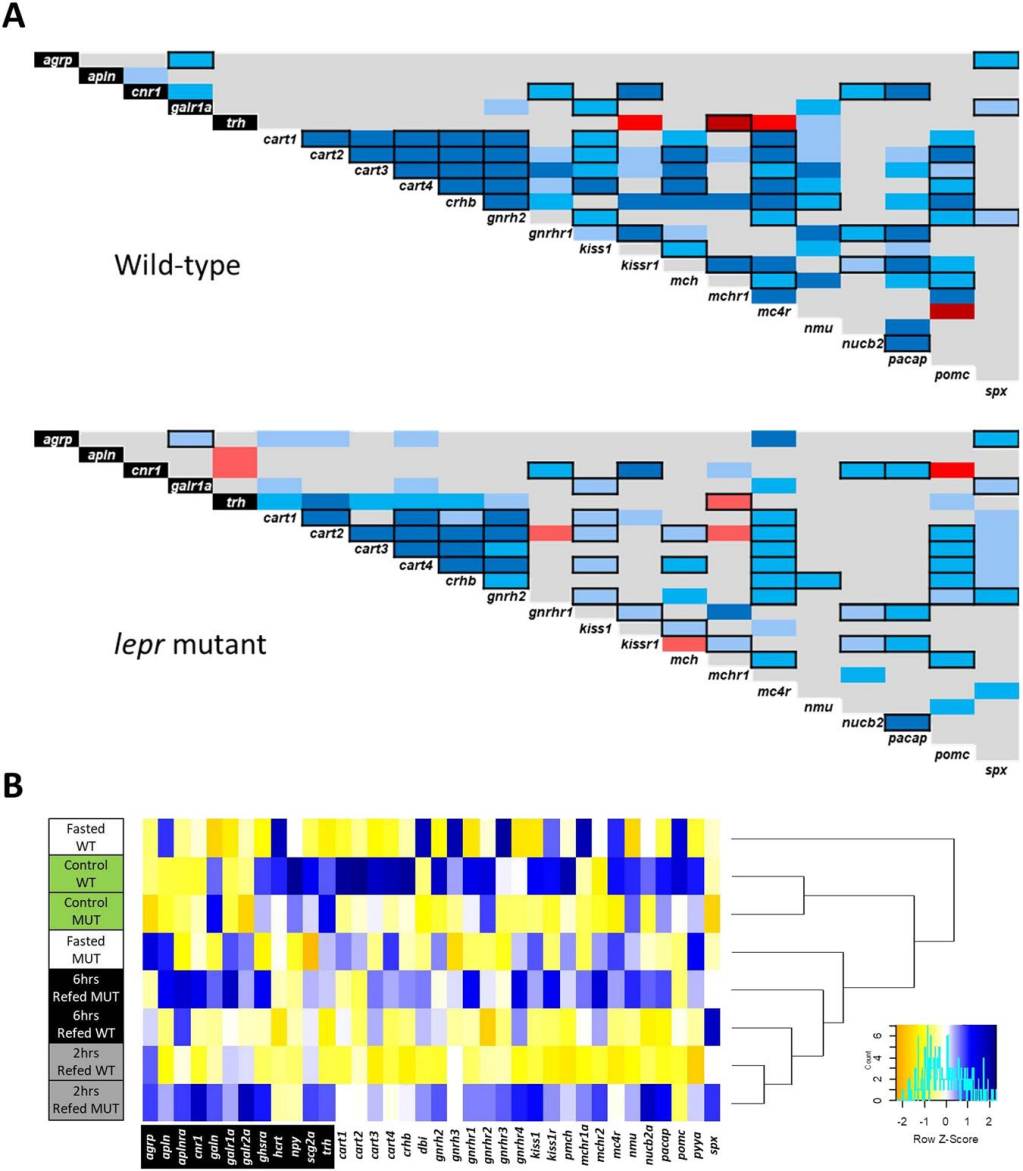
Expression correlations of appetite-regulating genes and clustering of the experimental conditions based on the gene expression patterns. (**A**) Pairwise expression correlations between appetite regulating genes in the brain of wild-type and *lepr* mutant zebrafish in the fasting-refeeding experiment. The red and blue colours respectively indicate negative and positive Pearson correlation coefficients and their light to dark shadings show significant levels of *P* < 0.05, *P* < 0.01 and *P* < 0.001. The genes specified with black and white backgrounds represent orexigenic and anorexigenic genes, respectively, and pairwise correlations delineated with black borders are similar between the two genotypes. (**B**) Dendrogram clustering of the experimental condition in each genotype based expression pattern similarities of all the appetite regulating genes in this study (the blue and yellow indicate higher and lower expression level respectively).

For each of the genotype, we conducted pairwise expression correlation analysis of the appetite-regulating genes, which showed differential expression in previous steps, in order to predict possible regulatory connections between the genes^28^. Most of the identified correlations appeared to be positive in both genotypes; however, not all of the positive correlations were similar between the genotypes, indicating gain or loss of certain regulatory connections between the genes in defective leptin signal (Fig. 5A). Among the positive correlations, which were similar between the two genotypes (*i*.*e*. unaffected by leptin signal), we observed strong connections between *cart1*,*2*,*3*,*4/crhb/gnrh2/mc4r/pomc*, *cnr1/kiss1*, *nucb2/pacap*, *cnr1/pacap*, and *agrp/spx*. We found only one negative correlation, *trh/mchr1*, which showed similarity between the genotypes (Fig. 5A). The overall higher number of positive correlations between and within orexigenic and anorexigenic genes in zebrafish brain demonstrates that their expression regulations have more positive co-regulatory connections in different feeding conditions.

In addition, we also found strong pairwise correlations, which were specific to each genotype, implying that regulatory connections are influenced by leptin signalling. For instance, we observed several strong positive correlations, which were lost in the *lepr* mutant group compared to the wildtype group, including those between *cart1/cart3*, *cart3/gnrhr1*, *crhb/kiss1r*, *crhb/mch*, *crhb/mchr1*, *mc4r/kiss1r*, *mc4r/mchr1*, and *nmu* with *cart3*, *gnrhr1*, *mch* and *nucb2* (Fig. 5A). On the other hand, we found a few gains of strong positive correlation in *lepr* mutant, including those between *cart2/trh*, *gnrhr1/mchr1*, and *agrp/mc4r*. Furthermore, 3 negative expression correlations were lost in *lepr* mutant; *kiss1r/trh*, *mc4r/trh*, and *mc4r/pomc*, whereas 6 negative expression correlations were gained within the mutant group; *apln/trh*, *cnr1/trh*, *cart2/gnrhr1*, *cart2/mchr1*, *kiss1r/mch*, and *cnr1/pomc* (Fig. 5A). These findings revealed that impaired leptin signalling influences potential regulatory connections between appetite-regulating genes in zebrafish brain, particularly the connections involving genes like *cart3*, *cnr1*, *gnrhr1*, *kiss1r*, *mch*, *mchr1*, *mc4r*, *nmu* and *trh*. It should be noted that using these genes as input for a vertebrate protein interactome prediction tool (STRING v10, http://string-db.org/) demonstrates potential molecular interactions between products of *cart3*, *kiss1r*, *nmu*, *trh*, *mc4r*, *pomca*, *agrp* and *mchr1a* in zebrafish and/or other vertebrates (Supplementary Fig. 1B).

Finally, we also preformed hierarchical clustering of the experimental groups, for both genotypes, based on the combined expression patterns of all appetite regulating genes in this study, in order to identify their similarities and divergences affected by leptin signal. Interestingly, we found that the refeeding groups were clustered distantly from the control groups, while the two genotypes were closely branched for control and refeeding groups (Fig. 5B). However, we observed distant clustering of the fasting groups, i.e. the wild-type fasting group was more similar, in overall expression pattern, to the control group, whereas the mutant fasting group was closer to the refeeding groups (Fig. 5B). Therefore, the brain expression of appetite-regulating genes shows the strongest divergence during fasting between wild-type and *lepr* mutant.

## Discussion

The main goal of this study was to explore the effects of impaired leptin signalling on transcription of appetite regulating genes in zebrafish brain. We compared expression profiles of known appetite-regulating genes in teleost fish in the brain of zebrafish with normal and non-functional leptin signalling, and provide evidence that both orexigenic and anorexigenic genes are influenced by leptin signalling. Among the orexigenic genes, we only found two genes, belonging to the same signal, *apln* (encoding apelin), and its cognate receptor, *aplnra*, to be differentially expressed in wild-type and *lepr* mutant, respectively. However, the changes seemed to differ in pattern between the two genotypes. *Apln* encodes a peptide that functions as an endogenous ligand for the G-protein coupled apelin receptor (*aplnra*), which in turn activates different tissue specific signalling pathways with mainly metabolic effects^29^. The observed difference was due to the increase in expression of *apln* during fasting in wild-type and expression induction of *aplnra* immediately after refeeding in the brain of *lepr* mutant (Fig. 1). The orexigenic function of *apln* has been demonstrated in Cypriniformes^30–32^ and Characiformes^33,34^. However, the orexigenic role of *apln* in zebrafish and the regulatory connection between leptin signalling and *apln*-*aplnra* in the brain have not been investigated. In goldfish, the direct administration of leptin appeared to have no effects on *apln* expression in the brain during normal feeding conditions^17^. In mice, a regulatory connection between leptin receptor and apelin has been already described; non-functional leptin receptor mutant (*db/db* mice) exhibit up-regulation in *Apln* expression^35^. We did not find expression difference between the two genotypes in the control group; however, the difference emerged after fasting and in the refeeding groups. The induction of *apln* by fasting in the wild-type group indicates its potential orexigenic effect in zebrafish, which seems to be weakened by the impaired leptin signal. On the other hand, we found *apln* to have higher expression in the *lepr* mutant than the wild-type during refeeding (Fig. 2). This suggests potential opposing role of leptin signal in regulating *apln* expression, under different feeding conditions, i.e. inducing effect during fasting and suppressive during refeeding. Future studies are required to elucidate the regulatory connections between leptin signalling and *apln*-*aplnra* in zebrafish under different feeding conditions.

In addition to *apln*, we found another gene encoding an agouti related neuropeptide, *agrp*, to have increased expression in the *lepr* mutant during fasting and 2hrs refeeding (Fig. 1). In mice, brain expression of *Agrp* is inhibited by leptin signal and its expression is mainly induced during fasting as well^36^. The comparison of the two genotypes also revealed higher *agrp* expression in the *lepr* mutant during fasting suggesting a suppressive role of leptin signal on *agrp* expression during fasting in zebrafish (Fig. 2). In vertebrates, *agrp* is mainly expressed in the hypothalamus and acts as antagonist for the melanocortin receptors, *mc3r* and *mc4r*^5^. We found a gain of positive expression correlation between *agrp* and *mc4r* in the *lepr* mutant (Fig. 5A), and this could be due to interference of functional leptin signal in co-regulation of *agrp* and *mc4r* in the wild-type zebrafish. In Cypriniformes, including zebrafish, *agrp* has been shown to have orexigenic effects^37–40^, and its overexpression can lead to obesity and higher growth rate in zebrafish^38^. In medaka, knockout of *lepr* had induced *agrp* expression indicating suppressive effect of active leptin signal on *agrp* expression and a conserved leptin-dependent negative regulation of *agrp* across the two distantly related fish species^41^. These effects might imply an anorexigenic role of leptin signal during fasting in zebrafish through suppression of *agrp* expression.

We found that two receptors of galanin, *galr1a* and *galr2a*, showed increased expression during refeeding in the mutant (Fig. 1), but in the direct comparison between the genotypes only *galr1a* showed higher expression in the mutant during fasting (Fig. 2). Studies of Cypriniformes suggest an orexigenic role of galanin signal in central nervous system^42–44^, and fasting induces the expression of galanin receptors in zebrafish brain^44^. Therefore, the increased *galr1a* expression during fasting in *lepr* mutant might reflect stronger appetite stimulation, through galanin signal in zebrafish with the impaired leptin function. Similar to *agrp*, the expression suppression of *galr1a* by leptin signal during fasting suggests yet another evidence for potential anorexigenic role of leptin signal during fasting in zebrafish. Two other genes, *cnr1* and *trh*, showed increased expression in the mutant at 2hrs and 6hrs refeeding groups, respectively (Fig. 1). The first gene encodes cannabinoid receptor 1, which appeared to have orexigenic effects in Cypriniformes^45–48^. A fasting experiment in zebrafish has shown that *cnr1* acts as an upstream regulator of *cart3* (an anorexigenic factor) in the nervous system, and is essential for suppression of *cart3* expression during fasting^45^. We did not find expression correlation between *cnr1* and *cart3* in both genotypes, but we found a gain of negative expression correlation between *cnr1* and *pomc* in the *lepr* mutant (Fig. 5A). Interestingly, *cnr1* has been shown to increase feeding through a POMC-related mechanism in rat hypothalamus^49^. Furthermore, blockade of *cnr1* restored leptin signaling in a leptin resistance mice through POMC/MC4R pathway^50^. Although such regulatory connections have not been shown in fish, our data suggest a potential leptin-dependent regulatory connection between *cnr1* and *pomc* in the zebrafish brain. The brain expression of *POMC* in mice is directly dependent on the function of leptin signal and its expression is reduced in the non-functional leptin mutant (*ob/ob* mice)^51^. Among teleost fish, in both Salmoniformes and Cypriniformes, leptin increases *pomc* expression in the brain^52,53^, and in medaka, knockout of *lepr* decreases *pomc* expression in the brain as well^41^. These findings are consistent with our results of *pomc* expression, which shows expression reduction in the *lepr* mutant at the normal feeding condition. Furthermore, the expression suppression of *cnr1* by leptin signal during 2hrs refeeding indicates potential anorexigenic role of leptin signal at early refeeding in zebrafish.

The second gene, *trh*, encodes thyrotropin-releasing hormone and only in goldfish it has been shown that *trh* can increase feeding and locomotor behaviours^54^. In addition to higher expression of *trh* in the *lepr* mutant at 6 hrs refeeding (Fig. 1), we observed several gains and losses of expression correlations between *trh* and other appetite-regulating genes, in the impaired leptin signal group. Among them were gains of positive expression correlations with *cart* genes. It is already known that *trh* acts as an upstream regulator of *cart* genes in goldfish hypothalamus^54^. Altogether, our findings demonstrate more active transcription of several orexigenic genes during fasting and refeeding in the brain of zebrafish with impaired leptin signal, as well as several potential leptin-dependent regulatory connections between these genes. This suggests that the anorexigenic role of leptin signals during changes in feeding conditions appears to be mediated through expression suppression of certain orexigenic genes.

Among the anorexigenic genes tested, we found distinct expression dynamics between the genotypes for almost all of the DE genes (except for *spx*). Perhaps the most striking findings about anorexigenic genes was the consistent and similar expression differences for all of the *cart* genes (Cocaine and amphetamine regulated transcripts) between the two genotypes. The analysis of expression dynamics for *cart1*,*2*,*3*,*4* genes revealed their reduced expression in the wild-type zebrafish, only in the treatment groups (Fig. 3). The direct comparisons of the two genotypes displayed higher expression of *cart* genes in the wild-type in the control group (Fig. 4). These results are very consistent with findings in rodents, in which not only leptin administration increases the brain expression of *Cart* gene^55^, but also the non-functional leptin signal leads to total absence of *Cart* expression in the brain^56^. Furthermore, our results demonstrate that changes in feeding conditions reduce the expression of all the *cart* genes in the brain. This is in agreement with the conserved anorexigenic role of *cart* genes shown in zebrafish^45,46^ and other teleost fishes^8^, although a previous study suggests that not all *cart* genes follow similar expression pattern in response to fasting^57^. We found that the reduced *cart* expressions in different feeding experiments were totally lost in the impaired leptin signal, suggesting leptin-dependent response of *cart* genes to different feeding conditions. In goldfish, it has been shown that *cart1* inhibition of feeding is regulated by leptin in the brain^58^, indicating a conserved regulatory connection between leptin signal and *cart* genes in both zebrafish and goldfish. Furthermore, we observed similar expression differences for another anorexigenic gene, *crhb* or corticotropin-releasing hormone^59,60^, could imply regulatory connections between *cart* genes and *crhb* in zebrafish brain. In particular, we found positive expression correlations between *crhb* and all *cart* genes in both genotypes (Fig. 5). In mammals and birds, it had been already shown that *CRH* is a downstream target of *CART* in the brain^61,62^, and in chicken *CART1* induces the *CRH* expression in the brain^63^. Additionally, *CRH* has been shown to be a downstream target of leptin in rat, and its brain expression increases after leptin administration^64^. These findings suggest anorexigenic role of leptin signal in the normal feeding condition through expression induction of anorexigenic *cart* and *crh* genes. Future studies are required to unravel regulatory hierarchy of *crhb*, *cart* genes and *leptin* signalling, in zebrafish brain, through identification of related GRNs (gene regulatory networks)^28,65^.

The only gene we found to have similar changes in both genotypes was *spx*, with increased expression during refeeding, however, in the direct comparisons of the genotypes it showed higher expression in the wild-type, for the control and 6 hrs refeeding groups. Spexin hormone encoding gene, *spx*, has been shown to have anorexigenic affects in the brain of Cypriniformes^66,67^. In goldfish, for instance, *spx* inhibits feeding, supresses the expression of *agrp* and *apln* while inducing the brain expression of *cart*, *pomc*, *mch* and *crh*^67^. A later study on zebrafish revealed that *spx* exerts its anorexigenic effects by suppressing *agrp* expression in the brain after feeding^66^. The anorexigenic effects of *spx* in fish might be complex and involve different regulatory mechanisms, as seen in a Perciforme species, in which *spx* acts only as a very short-term anorexigenic factor^68^.

A gonadotropin releasing hormone, *gnrh2*, and a receptor of the same signal, *gnrhr1*, were also among the DE candidate anorexigenic genes in our study. In both goldfish and zebrafish, *gnrh2* decreases food intake^69,70^, and in zebrafish overfeeding induces *gnrh2* expression in brain as well^69^. We did not find any expression changes for *gnrh2*, between the experimental groups, in both genotypes, although *gnrh2* and *gnrhr1* were the only ones showing differences in direct comparison of the genotypes (Fig. 4). Importantly, *gnrhr1* had lost many of its expression correlations with the other genes in the *lepr* mutant; in particular, *gnrhr1* lost its positive expression correlations with *cart* genes in the impaired leptin signal (Fig. 5). The *cart*-dependent regulation of *gnrh* secretion by activated leptin signal had been already reported in rat^71,72^. Also, in a Perciforme species, it has been shown that leptin increases the brain expression of *gnrh2*, but not *gnrh1*^73^. Our results indicate that the functional leptin signal induces the expression of anorexigenic *gnrh2* in zebrafish, in normal feeding condition. Interestingly, *gnrh2* is a direct downstream target of *crh* in goldfish^74^, and we also found similar expression pattern between *gnrh2* and *crh* in zebrafish, hence the expression induction of *gnrh2* by leptin signal can be through transcriptional activation of *crh* in zebrafish brain. It has been shown that anorexigenic effects of *gnrh2* are mediated through the receptor encoded by *gnrhr1*^69^. Thus, the increased expression of *gnrhr1* in later feeding conditions in *lepr* mutant might be a compensatory mechanism for lack of gnrh2 protein, as a result of lower *gnrh2* expression in the mutant, at earlier condition of normal feeding. However, such a compensatory mechanism has not been reported for *gnrh-gnrhr* signal in zebrafish.

We found another functionally connected group of anorexigenic genes, showing differential expression between the genotypes, including melanin concentrating hormone gene (*mch/pmch*) and its receptors (*mchr1*,*2*). The ligand encoding gene, *mch*, showed reduced expression in the treatment groups than the control group but only in the wild-type, whereas its receptors showed increased expression in the refeeding groups compared to the control group in the mutant (Fig. 3). However, in the direct comparison of the genotypes, *mch* and *mchr1a* showed higher expression in one of the refeeding groups in the *lepr* mutant than the wild-type (Fig. 4). In addition, both *mch* and *mchr1* had several gains and losses of expression correlations with other appetite-regulating genes, in the impaired leptin signalling zebrafish (Fig. 5). The appetite-regulating role of *mch* signal has not been investigated in zebrafish, but its anorexigenic role is already described in goldfish^75,76^. In mice, it is already shown that leptin signal acts as an upstream regulator of *MCH* and *MCHR1* in the brain^77,78^, and the absence of functional leptin signal it increases the expression of both genes in the brain^77^. Consistently, our results indicate that the expression of *mch* and its receptor is regulated by leptin signal in zebrafish; although future studies are required to understand their detailed regulatory connection with leptin signal, as well as their potential anorexigenic role in zebrafish.

Comparing the two genotypes, we also observed expression changes in genes belonging to different signals, including prepro-kisspeptin 1 and its receptor, *kiss1* and *kiss1r*, melanocortin 4 receptor, *mc4r*, neuromedin U preproprotein, *nmu*, nucleobindin 2 or nesfatin 1, *nucb2*, and pituitary adenylate cyclase activating polypeptide, *pacap*. Except for *kiss1* and *kiss1r*, which are only studied in sea bass (a Perciforme species)^79^, the rest of these genes are shown to have anorexigenic role in at least one Cypriniforme species, mainly goldfish^80–86^. Except for *nucb2*, for which the anorexignenic role already is described in zebrafish^84^, the transcriptional changes of the rest of the genes suggest their potential appetite-regulating role in zebrafish as well. In mice, leptin increases the brain expression of *Mc4r*^87^. Consistently, the zebrafish *lepr* mutant in our study shows reduced *mc4r* expression under normal feeding condition, indicating a conserved positive regulatory connection between leptin signal and *mc4r*. The potential regulatory connections between these genes and leptin singalling has not been studied in fish, but our results indicate the existence of such connections in zebrafish brain. In goldfish, *nmu* and *pacap*^81,88^, and in zebrafish, *nmu*^89^, act upstream of *crh* in the brain, and in this study, we also found positive expression correlation between *nmu* and *crh* in both genotypes, and between *pacap* and *crh* in the wild-type (Fig. 5), suggesting a similar conserved regulatory connection in the zebrafish brain. Another interesting example could be the losses of positive correlations between *nmu* and several other genes such as all of *cart* genes and *pacap* itself in the zebrafish with impaired leptin signalling. Altogether, these observations demonstrate the effects of leptin on the expression of several anorexigenic genes in zebrafish brain under different feeding conditions, as well as inter-dependent regulatory connections in normal functioning or impaired leptin signalling. Furthermore, the consistent lower expression level of several anorexigenic genes, in fish with impaired of leptin signal under normal feeding conditions, indicates that the potential anorexigenic role of leptin signal is mediated through these genes.

## Conclusions

The study provides evidence that both orexigenic and anorexigenic genes in the zebrafish brain are influenced by leptin signalling. We observed the most pronounced effect in the group of anorexigenic genes, where impaired leptin signalling resulted in reduced gene expression in several genes of the control group. This suggests a stimulatory effect of leptin on transcription of these anorexigenic genes in wild-type zebrafish under normal feeding conditions. We also observed effects of the impaired leptin signal on expression correlations between appetite regulating genes in zebrafish brain, implicating the existence of complex gene regulatory networks, which are under the influence of leptin signal. The study further indicates the potential appetite-regulating role of several of the investigated genes; however, additional studies are required to confirm the role of these genes in appetite regulation in zebrafish. Taken together, based on the expression changes of the appetite regulating genes, in the impaired leptin receptor signal transduction, we propose an anorexigenic role for leptin signalling in zebrafish. It should be emphasized, however, that all the observed transcriptional changes might not necessarily be translated into changes at the protein level. Furthermore, functional investigations, such as leptin-dependent phosphorylation of *Stat3* (a conserved leptin function between mammals and fish^90–92^), are useful to evaluate the level of impairment of leptin signal in the *lepr* mutant used in this study.

## Methods

### Fish breeding, fasting-refeeding scheme and sampling

In this study, we used zebrafish strain leptin receptor *sa12593*, obtained from European Zebrafish Resource Centre. The mutation was created by the Sanger Institute for the Zebrafish Mutation Project by replacing a cytosine with an adenine on chromosome 6, leading to the formation of a premature codon stop, causing an incomplete translation. For this study, we kept the *lepr* mutants and their sibling wild-type zebrafish in 3-liter recirculating tanks at 28.4 °C at the SciLife lab zebrafish facility at Uppsala University. The water parameters were checked regularly and maintained by facility staff members. We set up the dark-light conditions to 10 and 14 hours, respectively. Before the experiment, we fed the fishes with dry pellets once in the morning and with *Artemia* twice a day (middays and evenings). We divided a total number of 40 zebrafish of similar age, into four categories: fish fed by the facility as control group and fish fasted for a week; fish fasted for a week and sampled 2 hours after the refeeding or sampled 6 hours after the refeeding. Each group was subdivided into two categories: homozygote fish for the mutation on the leptin receptor and wild type fish, with 5 fish of each genotype per treatment group. It should be noted that within each group and its counterpart in the other genotype, we tried to include similar female/male ratios (1–2 females and 3–4 males) (Supplementary Data 1). We observed no significant differences in standard body length and net weight, as well as the hepato-somatic index (HSI) between the genotypes (Supplementary Data 1). Fasting resulted in a weight loss of around 10% in both genotypes. We conducted the feeding and sampling at similar time (during the day) within and between the genotypes, and sampling of the control groups was done 2 hours after feeding at the same time during the day. We anaesthetized the fish by immersion in a tricaine solution (MS-222) at a concentration of 0.4 mg/ml and euthanatized them by an immersion in an ice bath. Zebrafish were decapitated and each brain was carefully dissected and transferred into 200 μl of RNA*later*, an RNA extraction stabilization solution (Ambion Inc, Austin Texas), at 4 °C for one day and at −20 °C the next day until RNA isolation step.

### RNA isolation and cDNA synthesis

We isolated the RNA of sampled brains using Trizol (Ambion). The dissected brains were removed from RNA*later* (Ambion) and were put into a new 1.5 ml Eppendorf tube containing 200 μl of Trizol. Then, we homogenized the samples using a syringe needle (25 G Terumo needle and BD Plastipak 1 mL syringe). A quantity of 40 μl of chloroform (Sigma-Aldrich) was added, followed by room temperature incubation for 5 minutes. After incubation, we centrifuged the samples at 12 000 g/min for 20 minutes at 4 °C. The aqueous upper phase was carefully transferred into new RNAse-free microfuge tubes, and 1 μl of glycoblue (Ambion) and 100 μl of ice cold (−20 °C) isopropyl alcohol (Sigma-Aldrich) were added to the tubes. Again, we centrifuged the samples at 13 000 g/min for 20 minutes at 4 °C. The upper phase was removed and the RNA pellet was washed by adding 200 μl of ice cold (−20 °C) ethanol (VWR) at 75% mixed with DMSO solution, followed by centrifugation at 9 000 g/min for 5 minutes at 4 °C. Subsequently, a washing step was performed with 75% ethanol and repeated 3 times. The samples were then dried by opening the tube under a fume hood and solubilized by adding 10 μl of Nuclease-free water (Ambion). We performed a DNAse treatment on each sample to remove genomic DNA by Turbo DNA-*free* kit (Ambion) according to the manufacturer’s instructions. The quantity and quality of RNA was measured with a NanoDrop 1000 v3.7 (the absorption 260/280 nm ratios were all above 1.85) and 1000 ng of RNA input was used for the reverse transcription (RT). For the RT step, we used Superscript III First-Strand Synthesis System for RT-PCR (Invitrogen). In brief, the RNA samples were mixed with 0.5 μl of random primers (50 ng/μl) and 0.5 dNTP (10 nM), incubated at 65 °C for 5 minutes and then cooled down on ice for a minute. A mix composed of 2 μl of 5X First-Strand Buffer, 0.5 μl of DTT (0.1 M), 0.5 μl of RNase OUT (40 U/μl) and 0.5 μl of Superscript III RT (200 U/μl) was made and added to each sample. An incubation time of 5 minutes at 25 °C was set up, followed by an enzyme reaction time of 50 minutes at 50 °C and an enzyme inhibition time of 15 minutes at 70 °C. The final volume of 10 μl of cDNA was stored at −20 °C until the qPCR step.

### Candidate genes, designing primers and qPCR

To validate stably expressed reference gene(s), we selected 8 candidate reference genes based on studies of zebrafish, which had investigated suitable references genes across different experimental conditions, developmental stages and tissues^93–95^. As target genes, we selected 36 candidates involved in appetite regulation in Cypriniformes (mainly studied on goldfish and zebrafish) with the exception of *kiss1* and *kiss1r* which are only studied in sea bass (Perciformes) (Table 1). In order to design qPCR primers, we first obtain the gene sequences from Blastn, through a zebrafish database search engine (zfin.org)^96^. Then, we imported the sequences to CLC Genomic Workbench (CLC Bio, Denmark) and specified the exon/exon boundaries using annotated *Danio rerio* genome in the Ensembl database, http://www.ensembl.org ^97^. The primers with short amplicon sizes (<200 bp) were designed by Primer Express 3.0 (Applied Biosystems, CA, USA) and their dimerization and secondary structure formation were evaluated using OligoAnalyzer 3.1 (Integrated DNA Technology) (Supplementary Data 1).

For the qPCR step, 1 μl of diluted cDNA (1:20) of each sample was mixed with 7.5 μl of qPCR Master mix called PowerUp SYBR Green Master Mix (Thermo Fischer Scientific), 0.3 μl of forward and reverse primers (10 uM) and 6.2 μl of RNA-*free* water, for a total volume of 15 μl per tube. The qPCR instrument used was MxPro-3000 PCR machine (Stratagene, La Jolla, CA) together with MxPro software (Stratagene) for data mining. We conducted each biological replicate in two technical replicates for each gene and we followed a sample maximization method^98^ to have an optimal experimental set-up in each run. The reaction program was as follow: 50 °C for 2 minutes (1 cycle), 95 °C for 2 minutes (1 cycle), 95 °C for 15 second and 62 °C for 1 minute (40 cycles). A dissociation step (60–95 °C) was performed at the end of the amplification step. We estimated threshold cycles, number of copies and efficiencies by the software. To calculate primer efficiencies (E values), we used generated standard curves using serial dilution steps of pooled cDNA samples. For each gene, we ran standard curves in triplicates, and calculated the results using the following formula: E = 10[−1/slope]. R^2^ values were higher than 0.990 and efficiencies were ranging between 94.1–108.7% for all assays (Supplementary Data 1).

### Analysis of gene expression data

To identify the most suitable reference gene(s), we used three different software for calculating the expression stabilities; BestKeeper^99^, NormFinder^100^ and geNorm^101^. BestKeeper uses two algorithms for ranking of candidate reference genes; first one is based on the standard deviation (SD) of Cq values across all the samples and the second one considers expression correlations or BestKeeper index (r)^99^. NormFinder determines the most stable genes, through calculating expression stability values, which are based on analysis of inter- and intra-group variation in expression^100^. Finally, geNorm calculates mean pairwise variations in a stepwise manner between each gene and the other candidates (*M* value)^101^. The ranking outcomes, from each of these alone, cannot be reliable unless consistency of the results are observed between the software for the top ranked genes^102,103^. In this study, we used the Cq value of the most stable reference gene across both genotypes and different feeding conditions, Cq_reference_, to normalize Cq values of target genes for each sample (ΔCq_target_ = Cq_target_ − Cq_reference_). To calculate ΔΔCq values, we chose a biological replicate with lowest expression (highest Cq value) across all the samples for each gene (i.e. each gene can have its own calibrator sample), and then subtracted the ΔCq from the calibrator ΔCq value (ΔCq_target_ − ΔCq_calibrator_). We calculated the relative expression quantities (RQ values) through 2^−ΔΔCq^, and their logarithmic values (or fold changes) were used for statistical analysis^104^. We used student t-tests for the direct comparison gene expression levels of a target gene between wild-type and *lepr* mutant in each feeding condition. To analyse the expression dynamic of a target gene for each genotype, we implemented analysis of variance (ANOVA) test across the different feeding conditions followed by Tukey’s honest significant difference (HSD) post hoc tests. We used Benjamini-Hochberg procedure to correct for the false positive rate in the multiple comparisons^105^. Moreover, to identify the similarities in expression patterns between the target genes, we used pairwise Pearson correlation coefficients across the feeding conditions in each genotype. We implemented a dendrogram hierarchical clustering of expression values of the target genes to identify overall similarities between the feeding conditions and the genotypes. R (http://www.r-project.org) was used for all statistical analyses^106^. We explored the knowledge based interactions between the gene products by STRING v10 (http://string-db.org/), using zebrafish database for protein interactome^107^.

### Ethical approval

The fish handling procedures were approved by the Swedish Ethical Committee on Animal Research in Uppsala (permit C10/16). All methods were carried out in accordance with the guidelines and regulations of the Swedish Ethical Committee on Animal Research in Uppsala.

## Supplementary information

Supplementary Information.

Supplementary Information2.

## Data Availability

All the data represented in this study are provided within the main manuscript or in the supplementary materials.
