# Supplementary material for: Transcriptional study of appetite regulating genes in the brain of zebrafish (Danio rerio) with impaired leptin signalling
Source: Sci Rep. 2019 Dec 27;9:20166. doi: 10.1038/s41598-019-56779-z (PMC6934527; doi:10.1038/s41598-019-56779-z)
Supplement: Supplementary file 2 — Supplementary Information2. [file 41598_2019_56779_MOESM2_ESM.pdf]

Transcriptional study of appetite regulating genes in the brain of zebrafish (*Danio rerio*) with impaired leptin signalling

Ehsan Pashay Ahi[1],
Email: ehsan.pashay.ahi@ebc.uu.se

Mathilde Brunel[2],
Email: mathilde.brunel@slu.se

Emmanouil Tsakoumis[1]
Email : manolis.tsakoumis@ebc.uu.se

Monika Schmitz[1],
Email: monika.schmitz@ebc.uu.se

1. Department of Comparative Physiology, Uppsala University, Norbyvägen 18A, SE-75 236 Uppsala, Sweden
2. Department of Molecular Sciences, Swedish University of Agricultural Sciences, Allmas Allé 5, BioCentrum, Ultuna, Uppsala

Corresponding Author:
Monika Schmitz
Email: monika.schmitz@ebc.uu.se

A

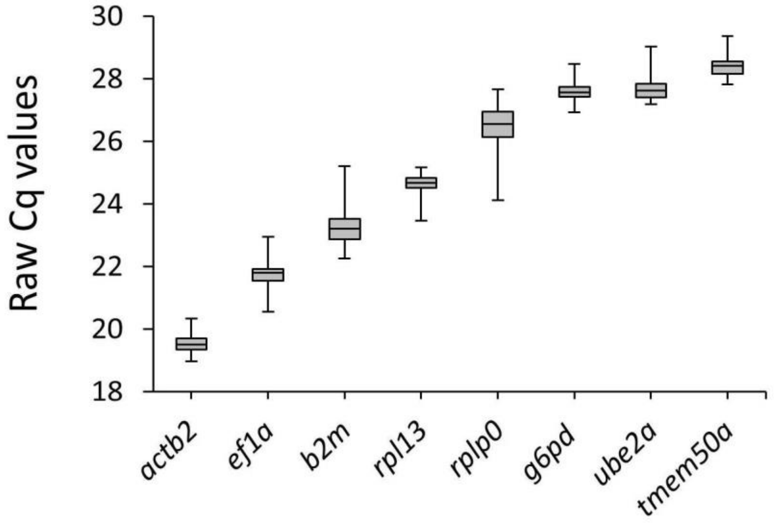

B

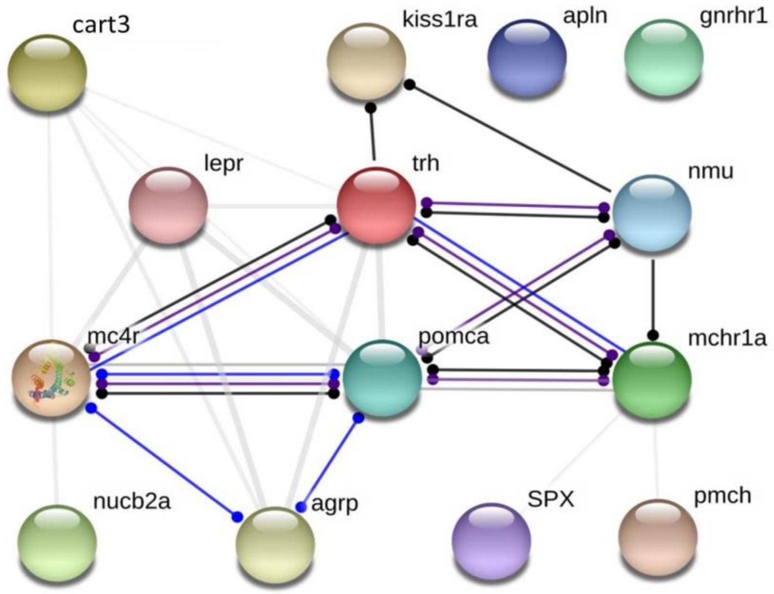

Supplementary Figure 1. (A) Raw Cq values showing expression levels of candidate reference genes in this study. The middle line and error bars denote the median and standard errors, respectively, and boxes indicate the 25/75 percentiles. (B) An interaction map predicting potential molecular connections between genes showing most divergence in expression correlations with other appetite-regulating genes in the impaired leptin signal (the map is created by a vertebrate protein interactome tool; STRING v10, http://string-db.org/).
